# The RavA-ViaA chaperone complex modulates bacterial persistence through its association with the fumarate reductase enzyme

**DOI:** 10.1016/j.jbc.2023.105199

**Published:** 2023-09-03

**Authors:** Vaibhav Bhandari, Sean E. Reichheld, Scott Houliston, Alexander Lemak, Cheryl H. Arrowsmith, Simon Sharpe, Walid A. Houry

**Affiliations:** 1 Department of Biochemistry, University of Toronto, Toronto, Ontario, Canada; 2 Molecular Medicine Program, The Hospital for Sick Children, Toronto, Ontario, Canada; 3 Princess Margaret Cancer Centre, University Health Network, Toronto, Ontario, Canada; 4 Department of Medical Biophysics, University of Toronto, Toronto, Ontario, Canada; 5 Structural Genomics Consortium, Toronto, Ontario, Canada; 6 Department of Chemistry, University of Toronto, Toronto, Ontario, Canada

**Keywords:** RavA, ViaA, MoxR, AAA+ proteins, von Willebrand factor type A domain, bacterial persistence, NMR structure

## Abstract

Regulatory ATPase variant A (RavA) is a MoxR AAA+ protein that functions together with a partner protein termed von Willebrand factor type A interacting with AAA+ ATPase (ViaA). RavA-ViaA are functionally associated with anaerobic respiration in *Escherichia coli* through interactions with the fumarate reductase (Frd) electron transport complex. Through this association, RavA and ViaA modulate the activity of the Frd complex and, hence, are proposed to have chaperone-like activity. However, the functional role of RavA-ViaA in the cell is not yet well established. We had demonstrated that RavA-ViaA can sensitize *E. coli* cells to sublethal concentrations of the aminoglycoside class of antibiotics. Since Frd has been associated with bacterial persistence against antibiotics, the relationship of RavA-ViaA and Frd was explored within this context. Experiments performed here reveal a function of RavA-ViaA in bacterial persistence upon treatment with antibiotics through the association of the chaperone complex with Frd. As part of this work, the NMR structure of the N-terminal domain of ViaA was solved. The structure reveals a novel alpha helical fold, which we name the VAN fold, that has not been observed before. We show that this domain is required for the function of the chaperone complex. We propose that modulating the levels of RavA-ViaA could enhance the susceptibility of Gram-negative bacteria to antibiotics.

Regulatory ATPase variant A (RavA) is a chaperone conserved across several pathogenic clades of the Gammaproteobacteria (1). In *Escherichia coli* and many other bacteria, the gene encoding for RavA is part of a two-gene operon along with the gene encoding for the protein von Willebrand factor type A interacting with AAA+ ATPase (ViaA). RavA belongs to the AAA+ family of ATPases, known for using the energy harnessed by hydrolysis of nucleotide triphosphates in remodelling molecular substrates (2, 3). Like other evolutionarily related proteins, interplay between RavA-ViaA can be described as an adapter-effector partnership (1, 4, 5).

As typical for many AAA+ proteins, RavA forms a hexamer. We previously solved the X-ray structure of *E. coli* RavA protomer (6) showing that the protein can be divided into three domains (Fig. 1*A*): a AAA+ domain, a β-barrel–like LARA domain, and a discontinuous triple-helical bundle that links the AAA+ domain and the LARA domains (Fig. 1*A*). The LARA domain mediates the interaction of hexameric RavA with the dodecameric lysine decarboxylase enzyme, LdcI, involved in the bacterial acid stress response (7, 8, 9, 10). Cryo-EM structures of RavA hexamer (10) and of the RavA–LdcI complex have also been obtained (8). Other groups have suggested that the LARA domain can also bind lipids (11), although we have not observed such interactions. Currently, there are no experimentally determined structures of ViaA. In previous work, based primarily on sequence conservation, we proposed that ViaA can be divided into at least two domains: a N-terminal ViaA domain (labeled as *previous NTV* in Fig. 1*B*; also see Results) and a C-terminal ViaA domain (CTV, Fig. 1*B*), which is a von-Willebrand factor A (VWA) domain. Biochemical studies showed that ViaA utilizes its VWA domain to mediate protein–protein interactions between RavA and its potential substrates (12). Along with acting as an adaptor for RavA, ViaA also enhances the ATPase activity of RavA (7, 11, 12).

**Figure 1:**
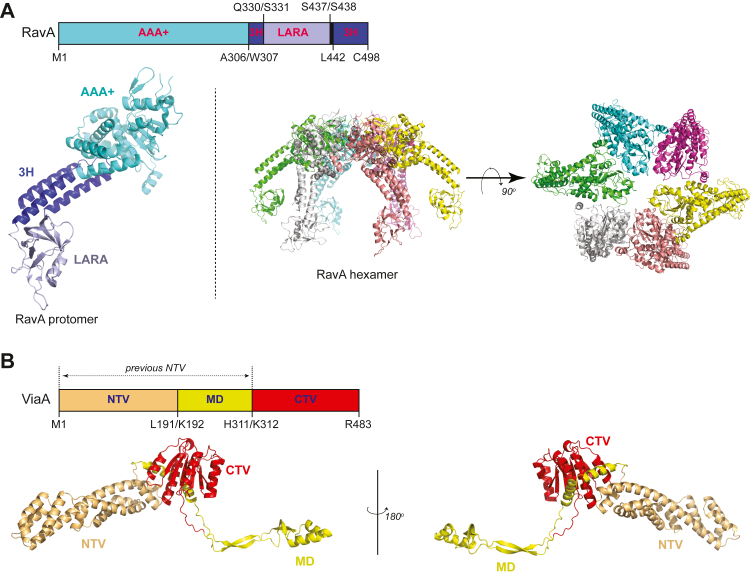
**Domain organization and structures of RavA and ViaA.***A*, *top* shows the domain organization of RavA. Domain boundaries are indicated along with the amino acid residues marking them. *Bottom* shows the structure of a RavA protomer (PDB ID: 3NBX), with the three domains colored in the same manner as the domain organization figure above, as well as of the RavA hexamer side (*left*) and top (*right*) views (PDB ID: 6SZA). All structures were drawn using PyMOL (https://pymol.org/2/). *B*, *top* shows the domain organization of ViaA. Domain boundaries are indicated along with the amino acid residues marking them. The term “*previous NTV*” refers to our definition of the NTV in our previous work (12). *Bottom* shows the predicted structure of full-length ViaA derived from AlphaFold (https://alphafold.ebi.ac.uk/entry/P0ADN0) with the domains colored in the same way as the domain organization figure above. RavA, regulatory ATPase variant A; ViaA, von Willebrand factor type A interacting with AAA+ ATPase; NTV, N-terminal ViaA domain.

In *E. coli*, RavA is mainly cytoplasmic while ViaA can localize to the inner membrane of the cell (11, 13), and both proteins have been linked to cellular respiration (12, 13). The very C-terminal helix in ViaA has been implicated in mediating interactions with phospholipids at the inner membrane (11). *In vitro*, we showed that RavA-ViaA repress the activity of the fumarate reductase (Frd) complex, an inner membrane–associated respiratory complex consisting of four proteins FrdABCD (12). FrdC and FrdD are membrane spanning subunits, while the flavoprotein FrdA and the iron–sulfur cluster–containing protein FrdB form the soluble part of the complex. We demonstrated that RavA-ViaA decrease the electron transport activity of the Frd complex because of a direct interaction between ViaA and FrdA (12); however, the functional significance of this interaction is not fully understood. Consistent with a potential role in regulating respiration and energy metabolism, the transcription of the *ravAviaA* operon in *E. coli* grown under aerobic conditions is primarily regulated by the stationary phase sigma factor, σ^S^, while under anaerobic conditions the operon is under control of the global transcription factor fumarate and nitrate reduction regulatory protein, Fnr, that controls the expression of a large number of target genes in response to anoxia (7, 12).

Adding to the complexity of RavA-ViaA cellular functions, we have previously shown that RavA associates tightly *via* its LARA domain with the inducible lysine decarboxylase, LdcI, to form a cage-like structure consisting of five RavA hexamers and two LdcI dodecamers (6, 7, 8, 14, 15). LdcI is involved in the bacterial acid stress response (16). The alarmone, ppGpp, binds and inhibits the activity of LdcI, but the formation of the RavA-LdcI cage prevents the binding of ppGpp to LdcI (6). Hence, RavA-ViaA might also have a role in the *E. coli* acid stress response.

To clarify the cellular role of RavA-ViaA, the influence of these proteins on Frd was further explored. Of particular relevance to this work are two prior significant observations: that (1) RavA-ViaA modulate the activity of Frd (12), and that (2) Frd is linked to antibiotic persistence (17, 18). With RavA-ViaA influencing Frd activity, we carried out experiments to determine the role of RavA-ViaA in the context of a persistence phenotype.

Antibiotic persistence describes the ability of a subpopulation of susceptible bacteria to tolerate antibiotic treatment without the acquisition of genetic markers conferring resistance (19, 20). Persistence may be an adaptive trait brought about by a confluence of genetics and epigenetics (21). It is a characteristic of the heterogeneity within a population and can emerge *via* different mechanisms (22, 23). A link between persistence and fumarate was observed when growth of *E. coli* in fumarate-supplemented LB media was shown to increase the frequency of persisters (17, 18). Increasing the levels of Frd led to a higher frequency of persisters (18). Furthermore, it was recently suggested that RavA-ViaA facilitate the uptake of aminoglycosides antibiotics across the inner membrane of *E. coli* under low-energy cellular states (24). In *Vibrio cholerae*, the deletion of *ravAviaA* was found to increase tolerance of the bacteria to aminoglycosides under aerobic conditions but this phenotype requires the presence of an envelope stress two-component system (25).

Our work has previously shown that ViaA utilizes its von Willebrand factor A domain (CTV) to facilitate the interaction of RavA with Frd complex through the FrdA subunit (7, 12). With the knowledge of an interaction between RavA-ViaA and FrdA (12) and the influence that Frd imparts on persistence of *E. coli* (18), experiments described below probed the potential significance of the RavA-ViaA proteins in the persistence phenotype. We identified conditions under which RavA-ViaA regulate Frd to abolish Frd’s enhancement of persistence. As part of this work, we also determined the structure of NTV using NMR spectroscopy, revealing a novel fold, which we named the VAN fold, that is required for the function of RavA-ViaA chaperone complex.

## Experimental procedures

### Construction of KO mutants

All strains used in this study are listed in Table 1. WT *E. coli* MG1655 was obtained from the American Type Culture Collection (catalogue number 700926). The *ravAviaA* KO (Δ*ravAviaA*) was previously constructed in our lab (12). P1 phage transduction method was used in the construction of additional KO strains (26). The antibiotic marker used to create the knockouts was then removed using the pCP20 plasmid that expresses the FLP recombinase (27) to obtain KO strains with no markers.

**Table 1:** List of strains, plasmids, and primers used in this study

Strain	Genotype	Reference
*Escherichia coli* MG1655	F-, rph-1, λ-	(51)
*E. coli* MG1655 Δ*frd*	*E. coli* MG1655 Δ*frdABCD*	Houry Lab
*E. coli* MG1655 Δ*ravAviaA*	*E. coli* MG1655 Δ*ravAviaA*	(12)
*E. coli* MG1655 Δ*frd* Δ*ravAviaA*	*E. coli* MG1655 Δ*frdABCD* Δ*ravAviaA*	This study


Plasmid	Description	Reference
p11	Cloning vector with ampicillin resistance marker	(52)
pETm60	Cloning vector with kanamycin resistance marker	(53)
pRavAViaA	p11-*ravAp*-*ravAviaA*, expression of RavA and ViaA under the control of the native promoter	(7)
pRavA_m_ViaA	p11-*ravAp*-*ravA*(K52Q)*viaA*, expression of the RavA Walker A motif mutant and ViaA under the control of the native promoter	(12)
pRavA	p11-*ravAp*-*ravA*, expression of RavA only under the control of the native promoter	(12)
pFrd	p11-*frdp*-*frdABCD*, expression of FrdABCD complex under the control of the native promoter	Houry Lab
pQlinkH	Cloning vector used for N-terminal His-tag fusion and overexpression	(54)
pETSUMO	Cloning vector used for N-terminal His_6_-SUMO solubility tag fusion and protein overexpression	(55)
p11-RavA	Expression plasmid for RavA with an N-terminal His_6_-tag followed by a TEV cut site	(12)
pETm60-AAA	Expression plasmid for N-terminal, 306 residue, AAA-domain (M1-A306) of RavA expressed as a fusion with the solubility enhancing NusA protein	This study
p11-RavAΔLARA	Expression plasmid for the RavA protein lacking the LARA domain (L336-A433). The construct is RavAΔLARA (M1-A335, L434-C498).	(6)
pETm60-CTV	Expression plasmid for the C-terminal domain of ViaA (K312-R483) expressed as a C-terminal fusion to the NusA protein	(12)
pETm60-ViaA	Plasmid used for the inducible expression of the NusA-ViaA fusion protein	(12)
p11-NTV311	Expression plasmid for the N-terminal, 311-residue segment of the ViaA protein (M1-H311). The construct contains a His_6_-tag followed by a TEV cut site at its N terminus	(12)
pQlinkH-NTV	Expression plasmid for the N-terminal, 191-residue fragment of the ViaA protein (M1-L191). The construct contains a His_6_-tag followed by a TEV cut site at its N terminus	This study
pETSUMO2-MD	Expression plasmid for the middle domain of ViaA (residues K192-H311) expressed with an N-terminal His_6_-SUMO	This study


Primer Name	Primer Sequence (5′ to 3′)
AAA-F	CCATCATATGGCTCACCCTCATTTATT
AAA-R	CGATGGATCCTTAGGCGTGACCGGTCATC
NTV-F	ATGCGGATCCATGCTAACGCTGGATACGC
NTV-R	CTGTAAGCTTTTATTAAAGCTGACCGGCGCTCAT
MD-F	ATGCGGATCCATGAAACGTGGCGACTATCAGT
MD-R	CTGTAAGCTTTTAATGTACCACCGGACGTTC

Abbreviation: TEV, tobacco etch virus nuclear-inclusion-a endopeptidase.

### Plasmids and expression vectors

Plasmid constructs used here were obtained from the Houry Lab and are referenced in Table 1. Protein expression vectors were constructed for the production of AAA+ domain of RavA (RavA residues M1-A306), NTV (ViaA residues M1-L191), and MD (ViaA residues K192-H311). All other protein expression constructs used in this study had been previously created at the Houry Lab. *E. coli* DH5α was used in the cloning of all constructs. The AAA-F and AAA-R primers (Table 1) were used to clone the AAA+ domain of RavA into the pETm60 vector. Similarly, the NTV-F and NTV-R primers (Table 1) were used in the cloning of the NTV of ViaA into the pQlinkH vector, while MD-F and MD-R (Table 1) were used for cloning the MD of ViaA into the pETSUMO2 vector.

### Protein expression and purification

The various RavA and ViaA constructs (Table 1) were produced using IPTG-inducible plasmids in BL21 (DE3) cell line. For each construct, 2 l to 6 l of BL21 (DE3) cultures were grown in LB media. The cultures were induced with 1 mM IPTG once they reached an absorbance (*A*_600_) of 0.4 units as measured using a Bio-Rad SmartSpec 3000 spectrophotometer. Cultures were then incubated at 18 °C for 16 to 20 h before being harvested and stored at −20 °C.

Protein expression constructs contained at the N-terminus His_6_-tag followed by a tobacco etch virus nuclear-inclusion-a endopeptidase (TEV) cut site, NusA-His_12_-tag followed by a TEV cut site, or His_6_-SUMO-tag as indicated in Table 1. Since the removal of the NusA tag from NusA-CTV severely reduced the solubility of the protein in aqueous buffer, the protein was used without removing the tag. Following Ni-nitriloacetic acid (Ni-NTA) affinity purification, proteins tagged with His_6_ or NusA-His_12_ were incubated with 0.1 mg/ml TEV protease, while those tagged with His_6_-SUMO were incubated with 0.01 mg/ml of ULP-1 protease. After protease incubation, protein samples were run through Ni-NTA affinity column a second time to separate the solubility/affinity tag fragments from the desired proteins as the tags were retained on the affinity beads. As the used TEV and ULP-1 proteases also contain H_6_-tags, they were also retained on the Ni-NTA beads. The resulting flow-through samples, containing the desired purified protein, were collected.

RavA, ViaA, *previous NTV* (ViaA residues M1-H311), and NusA-CTV were further purified using fast-purification liquid chromatography as follows. Ion exchange chromatography with Mono S 5/50 HR column (Cytiva) was performed to further purify RavA. ViaA protein was separated from traces of NusA using the Hi-Trap Heparin HP column (Cytiva). The Superdex 200 10/300 column (Cytiva) was used for the *previous NTV* and NusA-CTV constructs. Elution fractions were collected and visualized using SDS-PAGE to verify purity. All proteins were stored at −80 °C in buffer containing 50 mM Hepes (pH 7.5), 200 mM NaCl, 1 mM MgCl_2_, 1 mM DTT, and 5% glycerol.

### Trypsin digestion assay, EDMAN sequencing, and mass spectrometry

The 311-aa long ViaA fragment (M1-H311, referred to as *previous NTV*), was dissolved in incubation buffer (50 mM Hepes [pH 7.5], 300 mM NaCl, 1 mM MgCl_2_, 1 mM DTT). The protein was diluted to a final concentration of 1 mg/ml for digestion with trypsin. Commercially available bovine trypsin (Sigma-Aldrich) was dissolved in incubation buffer and added to a final reaction concentration of 20 μg/ml. The digestion sample was incubated at 20 °C for 24 h. Postincubation, a fraction of the sample was dissolved in Laemmli buffer and visualized *via* SDS-PAGE. Following SDS-PAGE, a prominent band was visible, estimated to be <25 kDa when compared to the protein molecular weight markers. The band was isolated from the SDS-PAGE gel and submitted to a commercial vendor for Edman sequencing of the first five amino acids.

A fraction of the remaining sample was assessed *via* MALDI-TOF for molecular weight determination. The dried-droplet method with sinapinic acid matrix was used to assess the protein’s molecular weight. Sinapinic acid was oversaturated into a mixture containing 30:70 (v/v) acetonitrile:water ratio and 0.1% trifluoroacetic acid. The solution was centrifuged for 1 min at 1000 rpm. The supernatant was collected. A 1 μl droplet of the collected solution was deposited onto a steel plate. One microliter of the posttrypsin-treated sample was mixed with the matrix solution. The sample was mixed with a pipette and allowed to air dry. Once the sample was dry, the steel plate was loaded onto the Bruker Microflex MALDI-ToF machine. The sample spectrum was obtained using a 20% laser power setting with a frequency setting of 200 Hz. FlexAnalysis software was used to generate the mass list and for spectrum analysis.

### ATPase assay

The ATPase activity for various constructs of RavA were tested using the ATP-NADH coupled assay as previously described (28). The reaction buffer was composed of the following components: 5 mM ATP, 0.2 mM NADH, 3 mM phosphoenolpyruvate, 4.7 U/ml pyruvate kinase, 7.4 U/ml lactate dehydrogenase, 5 mM MgCl_2_, 50 mM Hepes (pH 7.5), and 50 mM NaCl. Reactions were performed in triplicate, each in a volume of 150 μl in clear 96-well plate using the SpectraMax 340PC384 microplate reader. The absorbance at 340 nm was measured for a 10-min interval. The average and SD of the slopes were then calculated to obtain the ATP hydrolysis rate. The student’s *t* test was used to measure statistical significance and *p*-values < 0.05 were labeled as significant.

### Persistence assay

Frozen stock of the relevant *E. coli* strain was streaked onto an LB-agar plate. After incubation of the plates at 37 °C for 16 to 20 h, three colonies were picked and each was inoculated into 5 ml of LB media. The cultures were grown overnight at 37 °C. The overnight cultures were diluted 100-fold into 3 ml of fresh LB media. The cultures were shaken at 150 rpm at 37 °C for 2 h till they reached an *A*_600_ absorbance of 0.4, measured using Bio-Rad SmartSpec 3000 spectrophotometer. Once the desired *A*_600_ was reached, the 3 ml cultures were spun at 5000*g* for 5 min to collect the cells. Cells were resuspended into 3 ml of fresh LB media in the presence or absence of 30 μg/ml of kanamycin. Cultures were then grown for an additional 9 h. To perform colony counts, 1 ml of culture was spun down, washed with, and resuspended in PBS buffer. Five-fold serial dilutions were performed for each culture using pH 7.5 PBS. The diluted samples were then plated onto LB-agar plates and allowed to incubate at 37 °C for 16 h prior to counting of colony-forming units. For each strain/condition, three colonies were selected at random and grown in LB-kanamycin media. These colonies were verified to be susceptible to the antibiotic when inoculated in LB-kanamycin media. The numerical data for each strain represents three colony-level replicates derived from one bacterial stock. The data are presented as means and SDs. The student’s *t* test was used to measure statistical significance and *p*-values < 0.05 were labeled as significant.

### Structural determination of the NTV by NMR spectroscopy

The structure of the NTV (residues 1–191) was determined by NMR spectroscopy. NTV samples were prepared at concentrations ranging from 200 to 500 μM and buffered in 50 mM Hepes, pH 7.5, 100 to 200 mM NaCl, and 10 mM β-mercaptoethanol. Spectra were acquired at 313 K on Bruker spectrometers operating at 600 or 800 MHz, equipped with triple-resonance inverse cryoprobes. Initial partial resonance assignments were determined for ^15^N/^13^C-labeled protein using the automated FMCGUI method (29) based on a standard set of triple- and double-resonance NMR experiments collected as described previously (30). Resonance assignments were expanded and verified manually using CcpNmr analysis (31). All 3D spectra were acquired with nonuniform sampling in the indirect dimensions and were reconstructed by the multidimensional decomposition software qMDD (32), interfaced with NMRPipe (33). Peak picking was performed automatically and manually using CcpNmr analysis (31) and NMR-FAM-Sparky (34). Torsion angle restraints were derived using TALOS+ (35). H-bond restraints, when applied, were for residues unambiguously determined to be in secondary structural elements based on nuclear Overhauser effect (NOE) patterns, chemical shift assignments, and backbone torsion angles. Automated NOE assignments and structure calculations were performed using the software CYANA 2.1 (36) (https://cyana.org/wiki/Index.php/Main_Page). The best 20 of 100 CYANA-calculated structures were refined with CNSsolve (37) by performing a short restrained molecular dynamics simulation in explicit solvent (38). The final 20 refined structures comprise the NMR ensemble. Table 2 provides the structural statistics and quality scores. Data have been deposited in the respective databases PDBID: 7UGC and BMRBID: 31,004.

**Table 2:** NMR restraints, structural statistics, and quality scores for NTV

Design ID	NTV^a^
PDBID	7UGC
BMRBID	31,004
NMR restraints:	
Total nuclear Overhauser effects	2601
Intra-residual	731
Sequential (i – j = 1)	709
Medium-range (1 < i – j < 5)	888
Long-range (i – j ≥ 5)	503
Hydrogen bonds	38 × 2
Dihedral angles:	
*φ*	154
*ψ*	154
Structural Statistics:	
*r.m.s.d. from experimental restraints:*	
Distance restraints (Å)	0.019 ± 0.002
Dihedral angle restraints (°)	0.468 ± 0.085
*Violations in the NMR ensemble*^a^:	
Distance restraints (>0.5 Å)	0
Dihedral angle restrains (>5°)	0
*r.m.s.d. from idealized geometry:*	
Bond lengths (Å)	0.0147 ± 0.0002
Bond angles (°)	0.93 ± 0.013
Impropers (°)	1.73 ± 0.08
*Average pairwise r.m.s.d. (Å)*^b^:	
Heavy	1.65
Backbone	1.30
Structure quality scores:	
*Ramachandran plot (%)*^b^,^c^	
Most favored	94.5
Additionally allowed	5.4
Generously allowed	0.1
Disallowed	0
*Structural quality factors (raw/Z-scores)*^d^	
Procheck (phi/psi)	0.21/1.14
Procheck (all)	−0.04/-0.24
Molprobity clash	14.79/-1.00

a The NMR ensemble consists of the 20 lowest energy structures out of 100 calculated.

b Calculated for residues 2 to 166, inclusive.

c Based on Procheck analysis (56).

d Calculated using the PSVS server (https://montelionelab.chem.rpi.edu/PSVS/) (57).

The NMR ensemble structure for NTV was used as a query against the medium redundancy set of the MMDB on the publicly available Vector Alignment Search Tool (VAST) (39). VAST can be accessed at: http://www.ncbi.nlm.nih.gov/Structure/VAST/vast.shtml. The two highest scoring alignment structures are discussed.

## Results

### The binding of the middle domain of ViaA to RavA enhances RavA ATPase activity

RavA and ViaA are functionally linked proteins (12, 13). We had previously confirmed the interaction between RavA and ViaA indirectly by observing an enhancement of the RavA ATPase activity upon addition of ViaA (7). However, it remains unclear what regions of either protein are necessary to facilitate this interaction. Therefore, we attempted to map this interaction to discrete domains within each protein using ATPase activity assays.

RavA forms a hexameric complex with high ATPase activity (7, 10, 12). As mentioned in the introduction, based on the X-ray structure of RavA that we obtained previously (6), a RavA protomer can be divided into three domains: AAA+ domain, a discontinuous triple-helical bundle (3H), and a LARA domain (Fig. 1*A*). Unlike RavA, the structure for the ViaA protein has remained elusive. Based primarily on sequence alignment (12), ViaA can be divided into two main regions: an N-terminal region, containing the first 311 amino acids of the protein, which is not known to share similarity to any known protein, and a CTV consisting of residues 312 to 483 (Fig. 1*B*). However, structure prediction using AlphaFold (https://alphafold.ebi.ac.uk/entry/P0ADN0) (40), for example, suggests that ViaA can be divided into three domains (Fig. 1*B*): a highly alpha helical N-terminal domain, a flexible middle domain (MD), and a C-terminal VWA domain.

**Figure 2:**
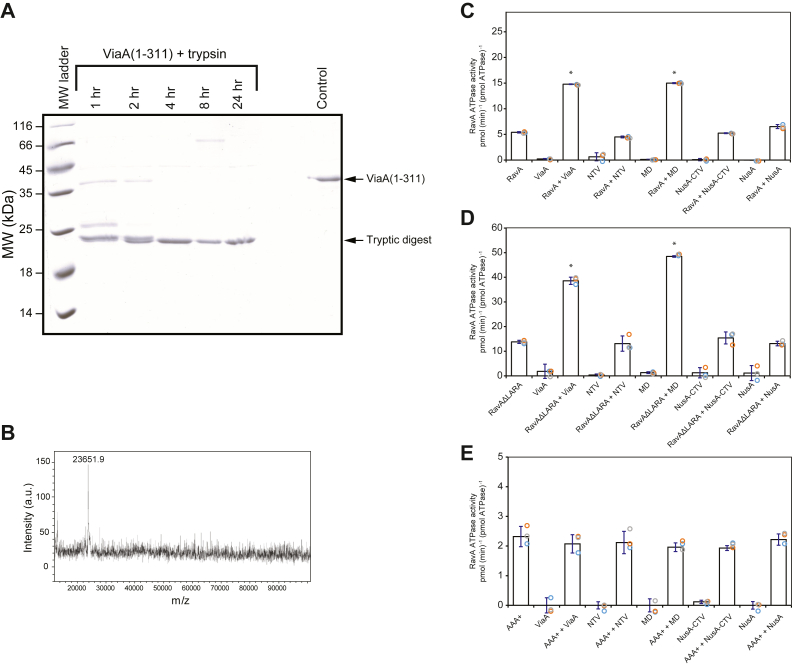
**Mapping the RavA–ViaA interaction.***A*, SDS-PAGE gel showing the 311-residue ViaA N-terminal segment on the *right* and the same protein fragment at different time points after it has undergone trypsin digestion. *B*, MALDI-TOF of the trypsin digested fragment (NTV, ViaA residues 1–191). *C–E*, ATPase activity for 0.5 μM of RavA (*C*), RavAΔLARA (*D*), or RavA-AAA+ (*E*) are shown in the presence of equimolar concentrations of various constructs of the ViaA protein. For all graphs shown, ∗ is indicative of significant changes in ATPase activity of RavA or RavA mutants upon addition of a given protein or domain with a *p*-value < 0.05. RavA, regulatory ATPase variant A; ViaA, von Willebrand factor type A interacting with AAA+ ATPase; NTV, N-terminal ViaA domain.

To further establish the domain arrangement of ViaA, the N-terminal 311-residue fragment of the protein was isolated and treated with trypsin. The trypsin digestion of the 311-residue fragment (∼35 kDa) led to the identification of a ∼23 kDa domain as shown on SDS-PAGE gel (Fig. 2*A*). The molecular weight of the generated fragment was confirmed by MALDI-ToF analysis (23.6 kDa; Fig. 2*B*). N-terminal EDMAN sequencing confirmed that the trypsin-stable domain spans residues 1 to 191 of ViaA. Therefore, in this work, we refer to residues 1 to 191 as the NTV, while residues 192 to 311 are referred to as the MD, and residues 312 to 483 as CTV (Fig. 1*B*). Note that, in our previous published work (12), NTV referred to residues 1 to 311 (labeled as “*previous NTV*” in Fig. 1*B*). Hence, we are redefining NTV in the current study.

Subsequently, the *in vitro* ATPase activity of RavA was tested in the presence of the different domains of ViaA. Figure 2*C* shows the results for the ATPase activity of full-length RavA protein in the presence of the three different ViaA domains. It should be noted that CTV was purified with an N-terminal NusA fusion protein as it could not be maintained in solution without the added solubility of the fused NusA. The NusA protein was purified and used as a control. There is a 3-fold increase in RavA ATPase activity in the presence of the full-length ViaA protein (Fig. 2*C*). When the isolated domains of ViaA were added, only MD led to an increase in RavA ATPase, similar to the effect of the full-length protein itself. NTV, NusA-CTV, and NusA had no effect on RavA ATPase (Fig. 2*C*). Therefore, MD appears to be the domain in ViaA responsible for the enhancement of RavA ATPase activity.

To further localize the region of interaction between the two proteins, two additional constructs of RavA were tested: RavAΔLARA (RavA lacking the LARA domain) and RavA-AAA+ (residues 1–306 of RavA) (Fig. 1*A*). Figure 2, *D* and *E*, show the ATPase activity for these constructs in the presence of the three ViaA domains. Unexpectedly, RavAΔLARA is more than 2-fold more active than the full-length protein (Fig. 2, *C* vs. *D*). This could be indicative of the potential physical constraints or autoinhibitory activity imposed by the LARA domain in the full-length RavA. Despite the overall increased activity, the effect of the ViaA domains on the ATPase activity of RavAΔLARA presents trends similar to the full-length protein. RavAΔLARA ATPase activity is enhanced by ViaA and by the MD of ViaA (Fig. 2*D*). In contrast, the ATPase activity of the AAA+ domain, which lacks the triple-helical bundle and the LARA domains, is not influenced by ViaA or its domains (Fig. 2*E*). Note that the AAA+ domain of RavA has about half the ATPase activity of the full-length protein (Fig. 2, *C* vs. *E*)

Collectively, the data of Figure 2 suggest that the MD of ViaA modulates the ATPase activity of RavA by associating with the triple-helical domain of the ATPase.

### RavA-ViaA suppress Frd-mediated bacterial persistence

While we currently have a good knowledge of the structure and interactions of RavA-ViaA, the exact cellular functions of this complex have remained elusive. This is partly due to the lack of a clearly observed phenotype upon the deletion of the respective genes. However, various experiments conducted by our group and others have suggested a possible role of this chaperone system in modulating *E. coli*’s as well as other Gram-negative bacteria’s sensitivity toward antibiotics (7, 13, 24, 25). In this regard, studies on the metabolic drivers of persistence in *E. coli* have implicated fumarate and Frd in increased persistence against antibiotics (18). It was suggested that the utilization of fumarate by Frd leads to increase in bacterial survival upon antibiotic treatment. Previously, we had shown that RavA-ViaA interact with and supress the ability of Frd to reduce fumarate to succinate (12); therefore, we became interested in determining whether RavA-ViaA may influence antibiotic persistence through Frd or if RavA-ViaA independently play a role in the process. For this purpose, we evaluated the persistence of various *E. coli* KO strains in the presence of fumarate.

**Figure 3:**
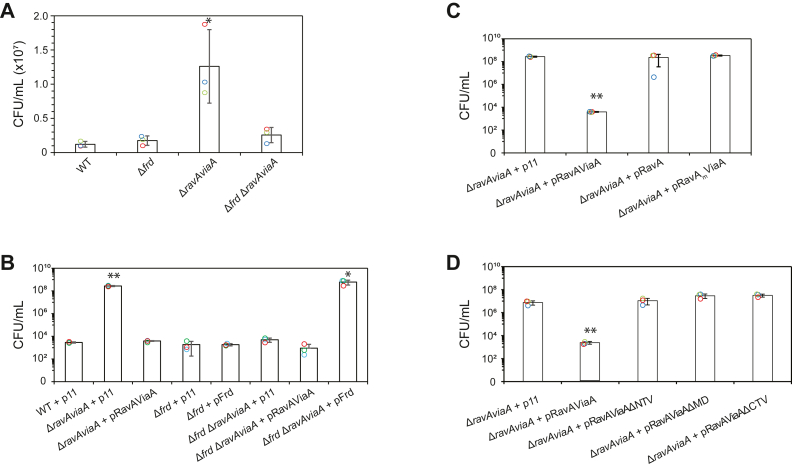
**Effect of RavA-ViaA on persistence.***A–D*, CFU counts for the indicated strains of *Escherichia coli* MG1655 grown in LB-fumarate media tested for persistence in the presence of 30 μg/ml kanamycin. For all the plasmids listed, the genes are expressed under their native promoter. p11, empty vector; pRavAViaA, p11-based construct expressing RavA and ViaA; pFrd, p11-based construct expressing FrdABCD; pRavA, p11-based construct expressing RavA; pRavA_m_ViaA, p11-based construct expressing functional ViaA but an ATPase-deficient K52Q mutant of RavA; pRavAViaAΔNTV, p11-based construct expressing RavA and ViaA deleted of NTV; pRavAViaAΔMD, p11-based construct expressing RavA and ViaA deleted of MD; pRavAViaAΔCTV, p11-based construct expressing RavA and ViaA deleted of CTV. For all graphs shown, ∗ and ∗∗ are indicative of counts that were found to be significant with a *p*-value < 0.05 and *p*-value < 0.01, respectively. ViaA, von Willebrand factor type A interacting with AAA+ ATPase; CFU, colony-forming unit; NTV, N-terminal ViaA domain; MD, middle domain; CTV, C-terminal domain of ViaA; RavA, regulatory ATPase variant A; Frd, fumarate reductase.

Deletion mutant strains were generated as needed (see Experimental procedures) and four strains were used in these studies: WT *E. coli* MG1655, a strain deleted of the FrdABCD operon (Δ*frd*), a strain deleted of the RavA-ViaA (Δ*ravAviaA*), a strain deleted of FrdABCD and RavA-ViaA (Δ*frd* Δ*ravAviaA*). All the deletion strains lack resistance markers (see Experimental procedures and Table 1). Persistence tests were performed using kanamycin in LB media with added fumarate (Fig. 3). Fumarate is used here as an additional carbon source. Kanamycin was selected for the tests as it was among the antibiotics against which Frd enzyme was shown to affect persistence (18). Also, RavA-ViaA have been shown to sensitize *E. coli* to aminoglycoside antibiotics (11, 13). Since kanamycin is most efficient against growing cultures, persistence levels during the early to mid-log phase of the cultures were measured. Therefore, the antibiotic was added when the cell culture had an absorbance *A*_600_ of ∼0.4. Colony counts were then measured 9 h after antibiotic addition (see Experimental procedures). All experiments were done at least in triplicates.

WT *E. coli* MG1655 strain was used to benchmark levels of cell survival across the deletion mutant strains. The absence of RavA-ViaA was found to increase persistence as compared to WT cells (Fig. 3*A*). The deletion of *frd* shows similar levels of persistence as WT (Fig. 3*A*). Importantly, though the deletion of *ravAviaA* increases persistence, the deletion of *frd* in addition to *ravAviaA* results in a strain having WT levels of persistence. Therefore, RavA-ViaA appear to play a negative role on the persistence of WT *E. coli* under these growth conditions. Furthermore, this influence of RavA-ViaA is observed only in the presence of the Frd enzyme, consistent with previous observations showing that Frd activity is reduced by the presence of RavA-ViaA (Wong *et al*., 2017).

To verify the importance of the interaction between RavA-ViaA and Frd in this observed persistence phenotype, complementation assays were performed (Fig. 3*B*). Plasmids expressing RavA-ViaA or Frd under their respective native promoters were introduced into mutant strains missing either one or both protein complexes. Mock expression plasmid p11 was also introduced into the strains as negative control. It should be noted that the use of p11 plasmid led to a decrease in the basal level of persistence for the WT strain. This is evident from the comparison between the persistence of WT strain in Figure 3*A* and WT + p11 in Figure 3*B*. This decrease was seen across all the strains containing the p11 plasmid except for the Δ*ravAviaA* strain that has enhanced persistence. While not further investigated, this could be due to the presence of the ampicillin resistance marker on the plasmid. The *frd* deletion mutant has near WT persistence and is not influenced by the introduction of Frd (Fig. 3*B*). The strain lacking RavA-ViaA is orders of magnitude more persistent than the WT strain. This increase in persistence is completely abrogated by the introduction of RavA-ViaA (Fig. 3*B*). This complementation is consistent with the observation that RavA-ViaA play a negative role in persistence. Finally, introduction of Frd but not RavA-ViaA into the triple mutant KO Δ*frd* Δ*ravAviaA* was sufficient to increase persistence in the absence of RavA-ViaA (Fig. 3*B*). These results clearly indicate that RavA-ViaA are acting on Frd to modulate persistence of the cells.

Next, an assessment of the importance of the RavA ATPase activity on the effect of RavA-ViaA in this fumarate-dependent persistence phenotype was performed (Fig. 3*C*). Initially, the Δ*ravAviaA* strain was complemented with one of the following plasmids: p11 (mock expression plasmid), pRavAViaA (expresses RavA and ViaA), pRavA (expresses RavA alone), pRavA_m_ViaA (expresses RavA ATPase–deficient mutant K52Q and WT ViaA). In all plasmids, the proteins are expressed under the endogenous promoter of the *ravAviaA* operon. While the expression of RavA and ViaA in Δ*ravAviaA* strain resulted in the reduction in persistence compared to that of Δ*ravAviaA* + p11 strain, the expression of RavA alone or RavA ATPase–deficient mutant with WT ViaA in Δ*ravAviaA* strain resulted in similar persistence level as that of Δ*ravAviaA* + p11 strain (Fig. 3*C*). These results demonstrate that the presence of ViaA and ATPase active RavA are required for this persistence phenotype.

Finally, to determine if all three domains identified in ViaA (Fig. 2) are required for the persistence phenotype, deletion mutants of ViaA domains were used. The Δ*ravAviaA* strain was complemented with RavA and ViaA or RavA and ViaA deleted of each one of its three domains (Figs. 1 and 2): NTV, MD, or CTV. As shown in Figure 3*D*, deletion of any one of the three domains of ViaA was sufficient to eliminate the functional complementation of RavA-ViaA on the persistence phenotype. MD of ViaA is required to bind RavA and CTV is required to bind FrdA. The exact function of NTV is not known, but we show here that it has an important role to play in the activity of the ViaA protein.

### The NMR structure of NTV shows a novel helical structure

Recent advances in the field of *in silico* protein structure prediction have been recognized to provide accurate models. The AlphaFold structure of full-length ViaA is shown in Figure 1*B*, with NTV, MD, and CTV highlighted by different colors. The model shows that NTV forms an alpha helical bundle that abuts the VWA domain in CTV, while MD forms a flexible domain that extends away from NTV. We propose that this would allow for the interaction of MD with the triple-helical bundle domain in RavA to enhance the ATPase activity of RavA (Fig. 1). Based on AlphaFold (Fig. 1*B*) and PrDOS (41) (Fig. S1) predictions, NTV of ViaA is connected to MD *via* helices, followed by a long unstructured region, while MD is connected to CTV *via* a long unstructured region. The extended nature of the structure and the length of random coils between the domains suggest that ViaA is a structurally flexible protein.

**Figure 4:**
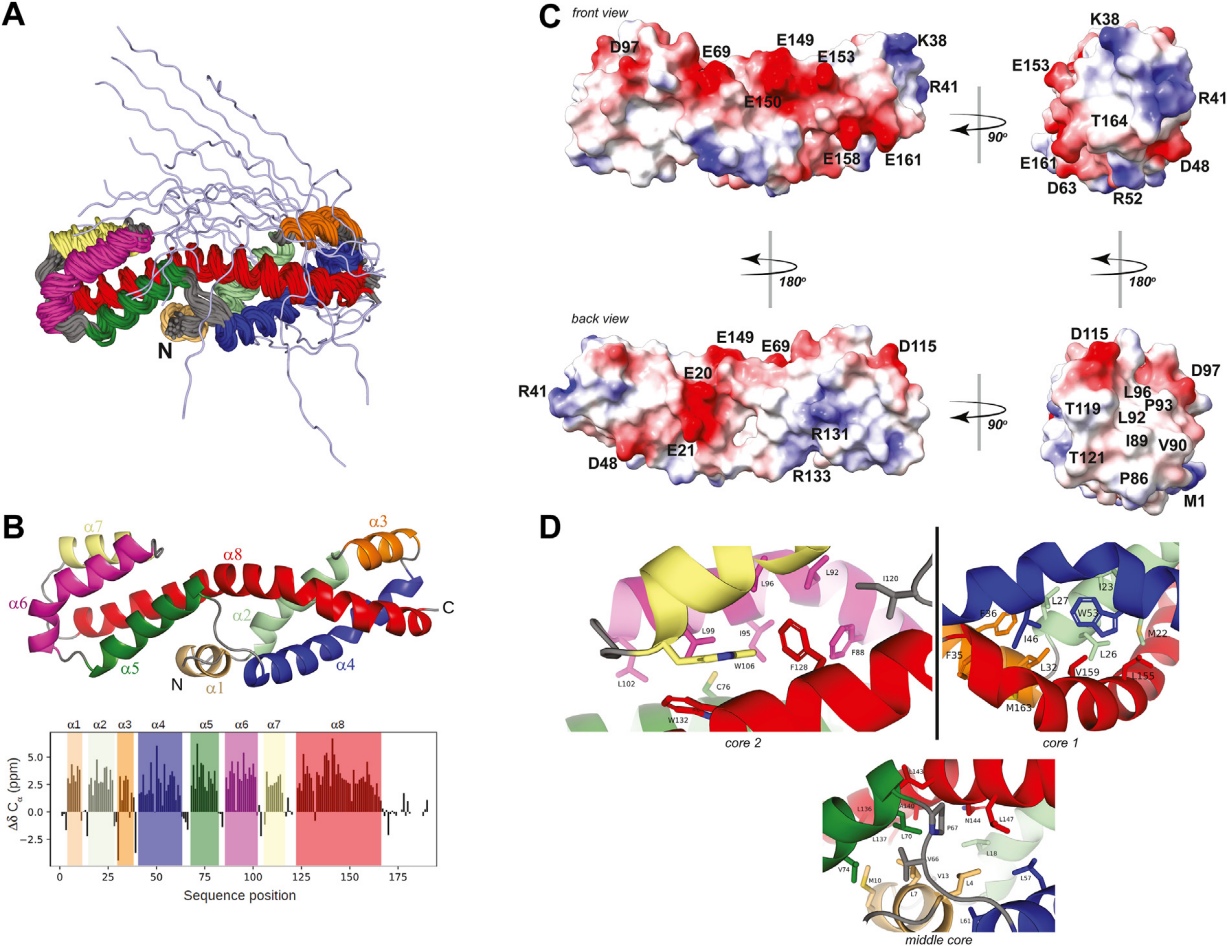
**Structure of NTV.***A*, shown is the NMR structural ensemble of 20 lowest energy conformations. Each helix is indicated by a different color. The disordered C terminus is shown in *light blue*. *B*, *top* shows the lowest energy conformation of NTV without the disordered C terminus. *Bottom* shows a plot of ^13^C_α_ secondary chemical shifts (difference from random coil) as a function of sequence position with location of helices highlighted with the same colors used in the structures. *C*, surface potential of NTV without the disordered C terminus in different views as indicated. The surface electrostatics were calculated considering the effects of solvation using the Adaptive Poisson–Boltzmann Solver (APBS) (49) plugin in ChimeraX (50). No counter-ions were added during this calculation. *D*, the three hydrophobic cores observed in NTV with core residues labeled as indicated. NTV, N-terminal ViaA domain.

In order to verify the predicted structure of ViaA, we initially attempted to obtain the X-ray structure of the full-length protein as well as of NTV+MD fragment of the protein. However, our efforts were not successful. Subsequently, we concentrated on obtaining the structure of NTV using NMR spectroscopy (see Experimental procedures and Table 2), which was successful. Shown in Figure 4*A* is the NMR-derived structural ensemble for NTV, which we deposited in the PDB as entry 7UGC. Figure 4*A* shows what we label as the front view of NTV. The domain forms a helical bundle composed of eight α-helices, which span residues M1-Q168 (Fig. 4*B*), while residues L169-L191 are disordered (Fig. 4, *A* and *B*). Fig. S2 shows the total NOEs/residue, the long-range NOEs/residue, and the random coil index as a function of sequence, calculated with Talos+ (35). All of the helices have residues with several assigned long-range NOEs (Fig. S2*B*), which define their relative position and packing within the tertiary structure. Each of the helices also have corresponding minima in the random coil index plot (Fig. S2*C*). NTV exhibits a novel fold that has not been observed before (see below) that we name the VAN fold for ViaA alpha helical N-terminal domain fold.

The surface of NTV exhibits both positive and negative surface potential (Fig. 4*C*). Interestingly, there is a negatively charged belt generated by glutamic acid residues 20, 21, 69, 149, 150, 153, 158, and 161 that runs around most of the middle of NTV. This might suggest a potential affinity to a positively charged surface of another protein or biomolecule, yet to be determined. On the other hand, there are far fewer positively charged sidechains available on the surface of NTV to form interactions with other molecules. Residues Lys38 and Arg41 (Fig. 4*C*), which are solvent exposed in both the NMR and AlphaFold structures, provide the highest concentration of positive charge on the surface of NTV. Another interaction site could be formed by a significantly hydrophobic groove formed by residues Pro86, Ile89, Val90, Leu92, Pro93, and Leu96 (Fig. 4*C*) on the solvent-facing end of NTV.

The core of NTV consists mainly of hydrophobic residues and can be divided into three main cores corresponding to the left, middle, and right parts of the protein (Fig. 4*D*). In core 2, there is a cysteine residue (Cys76) that forms sulfur–aromatic (S–π) interaction with Trp132 that has S to ring centroid distance of 5.5 Å inclined at an angle of 75° from the aromatic ring plane. This geometry of the interaction is generally consistent with previous observations on S–π interactions in the core region of protein structures. In such protein cores, the S–π interactions display a broad distance distribution of 4 to 11 Å and angles ranging from 0 to 90° (42, 43, 44).

**Figure 5:**
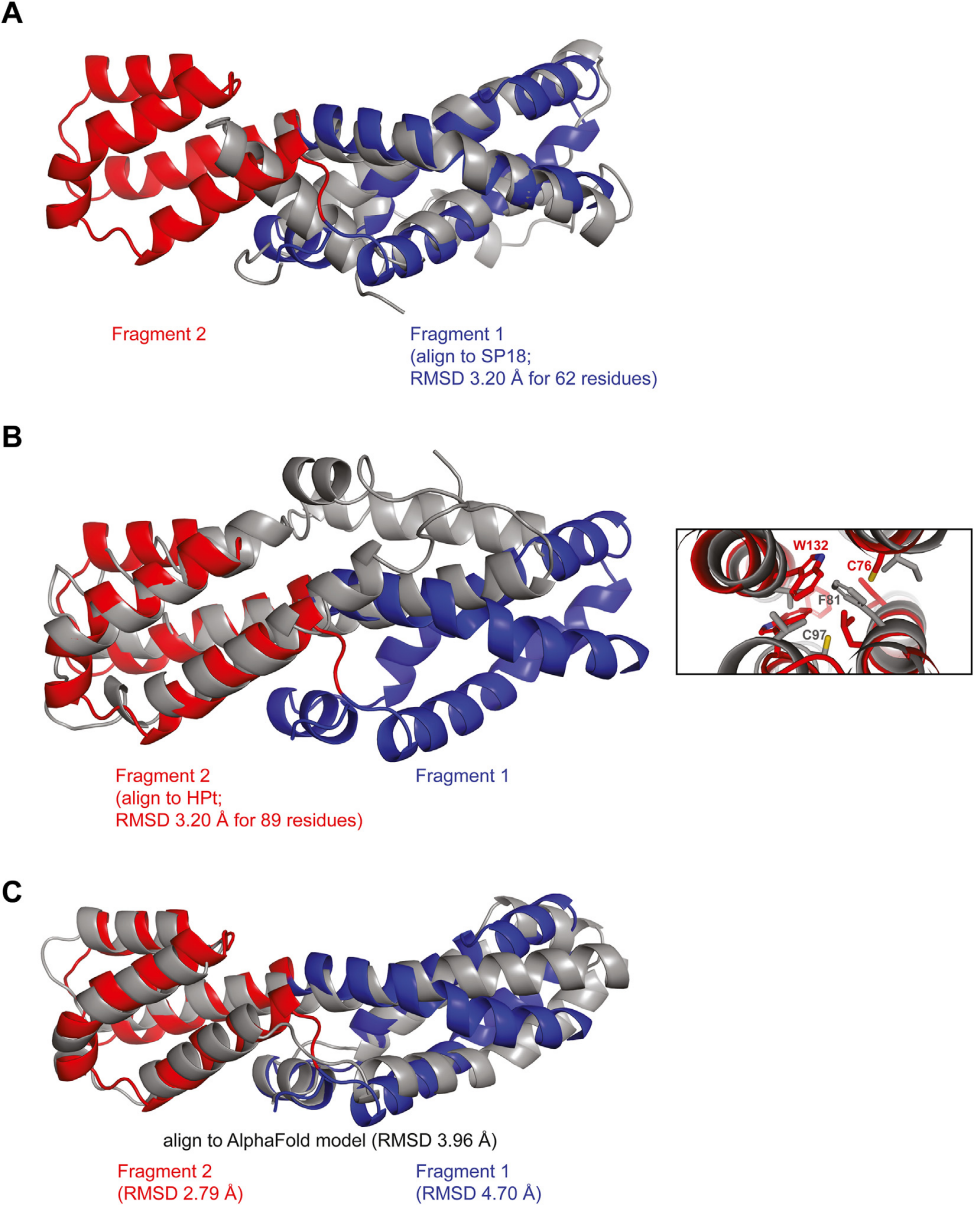
**Comparison of NTV structure with other structures.***A*, alignment of *top* VAST hit SP18 (PDB ID 1GAK, *gray*) (45) to fragment 1 of the NTV NMR structure (*blue*). The NMR structure of NTV is divided into two fragments based on VAST domain identification and structure comparisons. Fragment 1 (*blue*) is comprised of residues 1 to 65 and 140 to 165, while fragment 2 (*red*) spans residues 66 to 139. The RMSD value comparisons are given in the figures. *B*, on the *left* is shown an alignment of *top* VAST hit HPt (PDB ID 3US6, *gray*) (46) to fragment 2 of the NTV NMR structure (*red*). An overlay of the hydrophobic core around C76 in NTV with that of HPt around C97 is shown on the *right*. *C*, Cα alignment of AlphaFold structure of NTV (*gray*) to the solved NMR structure (*blue, red*). ViaA, von Willebrand factor type A interacting with AAA+ ATPase; HPt, histidine-containing phosphotransfer; NTV, N-terminal ViaA domain; VAST, Vector Alignment Search Tool.

No proteins with greater than approximately 20% sequence similarity to ViaA NTV were identified using the Protein BLAST search against the PDB or by querying the SWISS-MODEL homology-modeling server. Therefore, a 3D structure–based similarity search was carried out using the VAST (39), but no complete matches to the NMR-derived structural model of NTV were identified. However, VAST recognized two separate regions within NTV with structural similarity to other proteins. To avoid confusion with the separate domains that have been identified within full-length ViaA, we have denoted the VAST-identified regions as NTV fragment 1, corresponding to amino acid residues 1 to 65 and 140 to 165 (colored blue in Fig. 5) and NTV fragment 2 corresponding to residues 66 to 139 (colored red in Fig. 5). Using VAST, the highest match to fragment 1 was to a 62 residue fragment of SP18 protein of the marine snail Green Abalone (PDB: 1GAK; UniProt ID: Q25063) (Fig. 5*A*). The Sp18 is a sperm protein which, due to amphipathic properties, displays fusogenic abilities (45). For fragment 2, the highest match was an 89-residue fragment of the histidine-containing phosphotransfer (HPt) protein of the legume *Medicago truncatula* (PDB: 3US6; UniProt ID: B7FGU6) (46) (Fig. 5*B*). This protein is known to form complexes with protein partners and assist in phosphotransfer relays. Interestingly, Hpt has an unusually high fraction of Met and Cys residues with several of the Cys residues involved in S–π interactions (46). In Figure 5*B* right, an overlay of the hydrophobic core of NTV around C76 with that of HPt around C97 shows the presence S–π interactions in the core of both proteins, although the Cys residues do not overlap.

It is notable that the two closest structural matches for ViaA NTV are involved in mediating association with membranes or cofactor transport, both of these are aspects previously associated with the potential functions of ViaA (11, 12). ViaA associates with membrane-bound redox complexes, including Frd and NADH ubiquinone oxidoreductase (13). Furthermore, ViaA has been shown to localize to the inner membrane in *E. coli* (11). The combination of structural matches and the identified functional associations of ViaA infer potential functional roles for ViaA NTV in molecular mediation.

In Figure 5*C*, we compare the NMR structure of the first 168 residues of NTV against the AlphaFold model (https://alphafold.ebi.ac.uk/entry/P0ADN0). The NMR and AlphaFold structures show close backbone alignment throughout much of the length of the domain with the exception of residues lying at the very C terminal in NTV. The RMSD of Cα atoms is 3.96 Å for residues 1 to 167, with the strongest agreement between the two models in fragment 2, as exemplified by the lower RMSD value of 2.79 Å. The most salient differences between the two structures lie in fragment 1, which has an RMSD compared to the NMR structure of 4.70 Å (Fig. 5*C*). The models display a noticeable divergence in the direction of the bend at the midway point of helix α8, which runs through fragments 1 and 2 (see Fig. 4*B*). The difference in the orientation of helix α8 coincides with a disparity in the hydrophobic packing arrangement and relative orientations of the smaller helices α3 and α4. When the fragments 1 and 2 of the two models were aligned independently, the RMSD values were reduced to 3.31 Å and 1.23 Å, respectively, indicting greater similarity between the two fragments than the global alignment would suggest. The other significant difference between the two models appears in the region pertaining to residues 170 to 190, with AlphaFold of the full-length protein predicting this region to be alpha-helical, while the NMR-derived structure identified it as a random coil (Fig. 4, *A* and *B*). This could be due to the possibility that this region of NTV is stabilized by interaction with CTV (Fig. 1*B*). Hence, the structural model of NTV predicted by AlphaFold is generally consistent with the experimentally derived structure, with the most significant differences corresponding to the lowest confidence levels within the NTV domain of the AlphaFold model. It will be interesting to learn whether these differences highlight an artifact in the computational modeling, a structural influence imparted onto the purified domain separated from the rest of the ViaA protein, or a degree of flexibility inherent in the protein structure.

## Discussion

**Figure 6:**
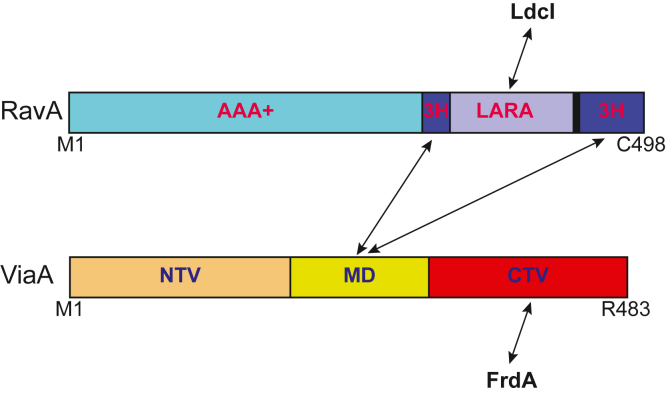
**The interactions of RavA and ViaA.** Domain organization of RavA and ViaA highlighting the domains mediating the interactions between the two proteins, as well as with LdcI and FrdA. Frd, fumarate reductase; LdcI, inducible lysine decarboxylase; RavA, regulatory ATPase variant A; ViaA, von Willebrand factor type A interacting with AAA+ ATPase.

In this work, we have further clarified the domain organization of ViaA and the interaction between RavA and ViaA. The results of Figure 2, *C*–*E* clearly demonstrate that the binding between MD of ViaA and the triple-helical bundle domain of RavA results in the enhancement of the ATPase activity of RavA (Figs. 6 and S3). Our efforts at solving the structure of full-length ViaA were unsuccessful, however, we were able to determine the structure of NTV using NMR approaches (Fig. 4). The structure reveals a novel helical fold, which we named the VAN fold, that might be responsible for promoting interactions between ViaA and other biomolecules (proteins, membranes, or DNA/RNA) that need to be determined to fully establish the molecular function of this domain.

It should be noted that the regions within Sp18 and HPt proteins that were found to be structurally similar to NTV are merely fragments of these proteins and cannot be considered as complete domains. NTV aligns to only 62 of 141 and 89 of 153 residues of Sp18 and Hpt, respectively. Furthermore, while VAST identifies fragments 1 and 2 as distinct domains, the NMR structure of NTV shows that these fragments are structurally connected by the middle core of NTV and the long-shared helix (α8) that runs through both fragments. It is unlikely that these two fragments would fold independently into their native structures without the support of these interconnected interactions. As such, we believe that the full-length NTV constitutes a distinct folding unit. Since no structurally similar matches to the folded region of NTV could be found in the PDB we propose that VAN is indeed a novel fold.

In investigating the cellular function of RavA-ViaA, the work performed here followed recent observations where fumarate and Frd were associated with increased persistence against antibiotics (18). Having previously shown that RavA-ViaA are interactors of Frd, we performed a series of experiments to identify if, and how, this interaction may be involved in persistence of *E. coli* against antibiotics. Results show that the presence or absence of RavA-ViaA can have a significant influence on the level of antibiotic persistence (Fig. 3).

In LB-fumarate media cultures with kanamycin, the presence of RavA-ViaA was found to have a negative effect on the number of persisters formed with the Δ*ravAviaA* mutant producing a higher number of persisters in comparison to WT (Fig. 3*A*). The persister counts not only increased for the Δ*ravAviaA* strain but returned to WT levels once Frd was also deleted in the Δ*frd* Δ*ravAviaA* knockout (Fig. 3*A*). The deletion of Frd produced a similar number of persisters as WT. Since we previously observed that RavA-ViaA inhibit the activity of the Frd enzyme (12) and that ViaA directly binds FrdA through its CTV (Figs. 6 and S3), these results suggest that the RavA-ViaA pair can influence persistence through their inhibition of the Frd enzyme *via* a direct physical interaction with FrdA.

It should be noted that our observations contradict the recent results of El Khoury *et al*. (24), who proposed that RavA-ViaA can facilitate the uptake of aminoglycosides under low-energy conditions, but that the proteins do not modulate Frd enzymatic activity, which could be attributed to different experimental setup. In our previous work (12), we clearly observed a direct interaction between ViaA and FrdA. Furthermore, in our current work, the observed persister phenotype was dependent on this interaction as a plasmid-expressing, ATPase-deficient RavA and WT ViaA (Fig. 3*C*) or a plasmid-expressing WT RavA and ViaA deleted of any one of its three domain (Fig. 3*D*) did not complement the persister phenotype of the Δ*ravAviaA* strain. Also, a plasmid-expressing WT RavA and WT ViaA did not complement the persister phenotype of the Δ*frd* Δ*ravAviaA* strain (Fig. 3*B*). These results clearly demonstrate that the persister phenotype depends on a direct interaction between RavA-ViaA and the Frd complex in LB-fumarate media. El Khoury *et al*. (24) used different conditions than those used in our experiments. Most of their experiments were carried out using LB-fumarate and gentamicin as the antibiotic rather than kanamycin. Furthermore, their assays measured cell killing rather than persistence; therefore, they used a different antibiotic treatment protocol than ours. This might explain the differences in our results.

Adding to the complexity of the RavA-ViaA function is the observation of the tight binding between RavA and LdcI, mediated by the LARA domain of RavA, to form a cage-like structure consisting of two LdcI decamers and five RavA hexamers (Figs. 6 and S3). The RavA–LdcI complex prevents the inhibition of LdcI activity by the alarmone ppGpp (6). Therefore, it can be suggested that under (1) acidic (2), growth-limiting (3), anaerobic, and (4) fumarate-rich environmental conditions resulting in the induction of the stringent response as well as the acid stress response, RavA-ViaA might prevent the inhibition of LdcI activity but inhibit Frd activity. Such environmental conditions might be encountered by Gram negative bacteria upon invasion of the host. Under these conditions, lysine would be metabolized, but the metabolism of fumarate would be reduced. In this regard, it is interesting to note that Radoš *et al*. (47) found many tricarboxylic acid cycle-derived amino acids including lysine to have their highest cellular concentrations in fumarate-fed cells. So, higher fumarate levels could lead to higher lysine levels. Therefore, based on the observations made in this work as well as the published literature, we propose that RavA-ViaA might play a critical role as a chaperone to balance the metabolome of the bacteria under specific stress conditions encountered upon bacterial infection of the host.

## Data availability

All data are presented in the article. Materials are available from the corresponding author upon reasonable request.

## Supporting information

This article contains supporting information (8, 10, 35, 41, 48).

## Conflict of interest

The authors declare that they have no conflicts of interest with the contents of this article.
