# Supplementary figures and images for: The RavA-ViaA chaperone complex modulates bacterial persistence through its association with the fumarate reductase enzyme
Source: J Biol Chem. 2023 Sep 3;299(10):105199. doi: 10.1016/j.jbc.2023.105199 (PMC10585395; doi:10.1016/j.jbc.2023.105199)

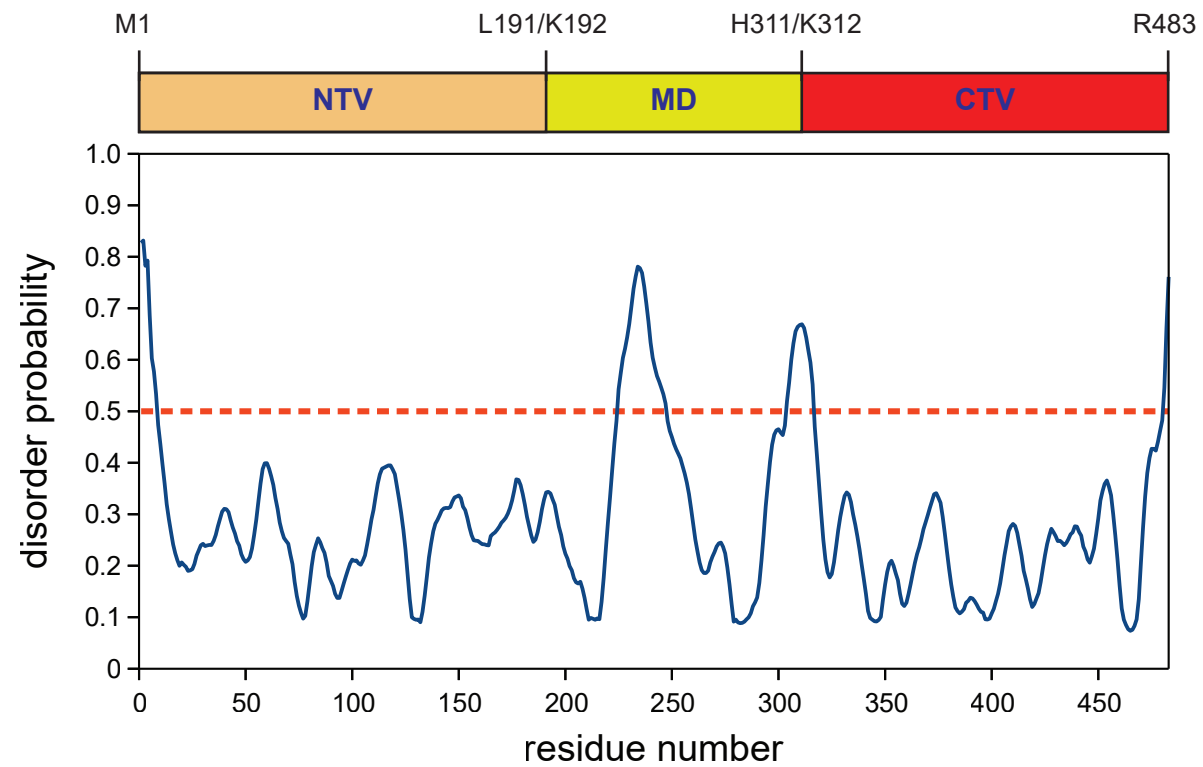

Supplement: Figure S1 [file mmc1.pdf]

A

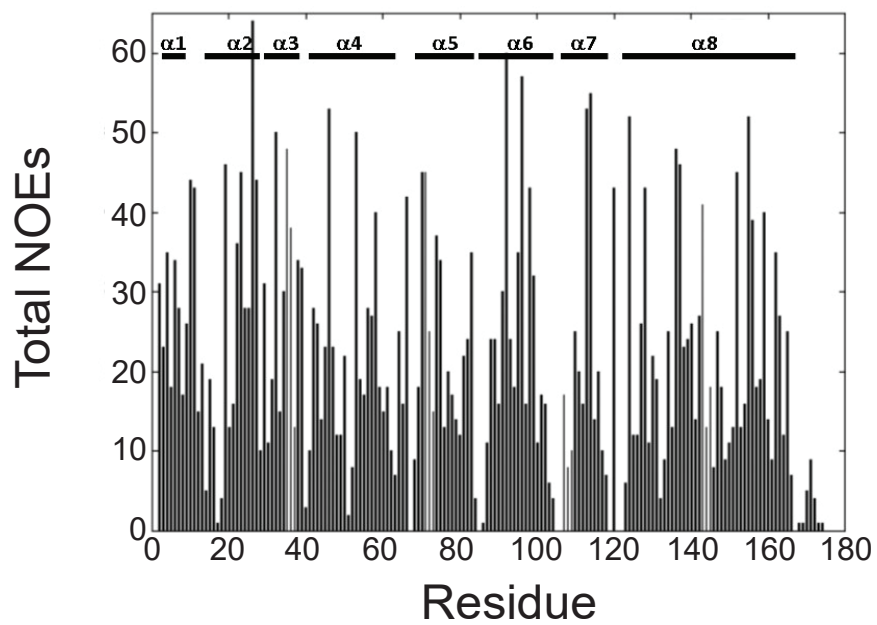

B

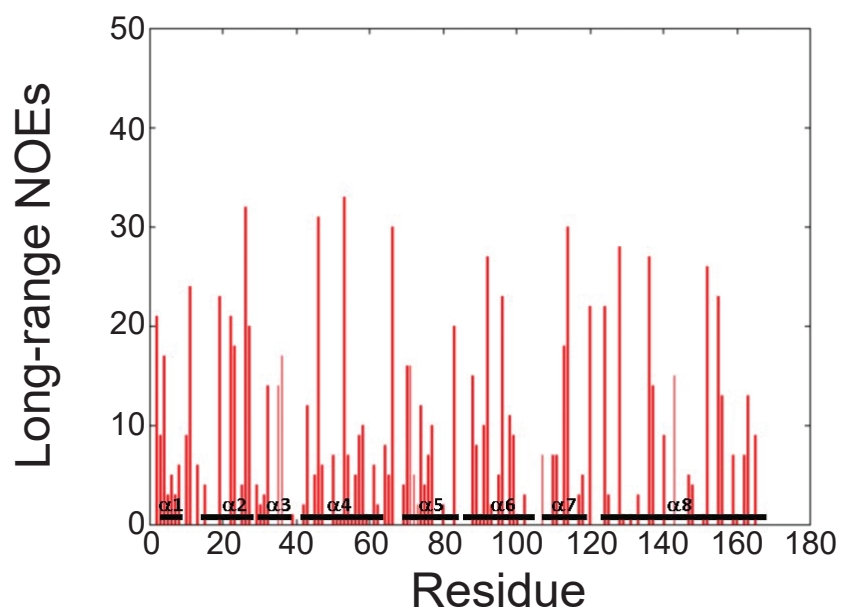

C

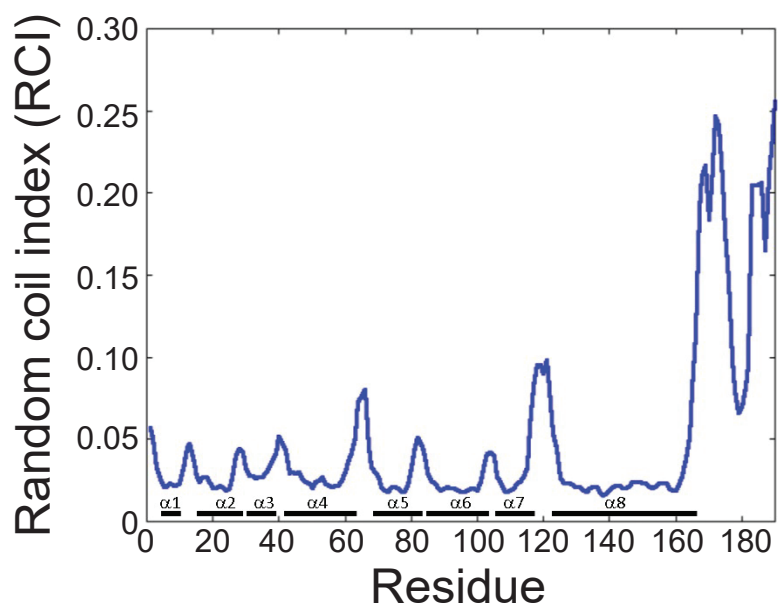

Supplement: Figure S2 [file mmc2.pdf]

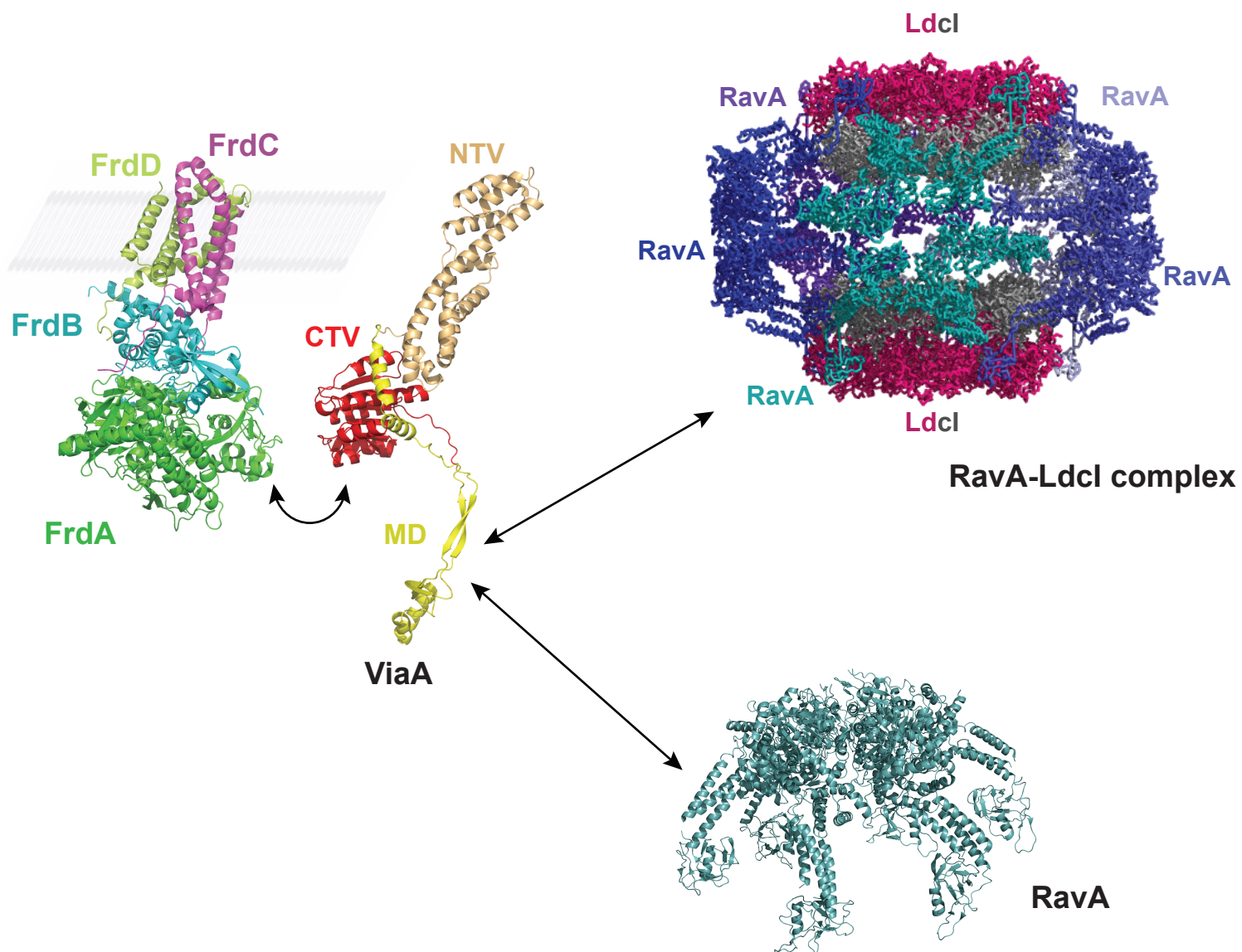

Supplement: Figure S3 [file mmc3.pdf]
