# Supplementary material for: The RavA-ViaA chaperone complex modulates bacterial persistence through its association with the fumarate reductase enzyme
Source: J Biol Chem. 2023 Sep 3;299(10):105199. doi: 10.1016/j.jbc.2023.105199 (PMC10585395; doi:10.1016/j.jbc.2023.105199)
Supplement: Supplementary Figure legends [file mmc4.docx]

**LEGENDS FOR SUPPLEMENTAL FIGURES**

**Figure S1. Prediction of disordered regions in ViaA.**

The PrDOS (1) disorder prediction probability as a function of sequence position is shown (blue line). Two regions within the MD, which connect this domain to the flanking NTV and CTV domains have a predicted disorder probability above the disorder threshold (dashed red line). Domain arrangement of ViaA is shown on top (see **Figure 1B**).

**Figure S2. Assigned NOEs per residue and the random coil index for the NTV structural ensemble.**

(A) The total assigned NOEs per residue for the NTV structural ensemble.

(B) The assigned long-range NOEs per residue for the NTV structural ensemble.

(C) The random coil index (RCI) as a function of sequence for the NTV ensemble, calculated using Talos+ (2).

**Figure S3. Schematic of the interactions of RavA and ViaA.**

Shown is a structural view of the known interactions between FrdA, ViaA, RavA, and RavA-LdcI complex using the X-ray structure of FrdABCD (PDB ID 1KF6) (3), AlphaFold Structure of ViaA (https://alphafold.ebi.ac.uk/entry/P0ADN0), cryoEM structure of RavA hexamer (PDB ID 6SZA) (4), and cryoEM structure of RavA-LdcI complex (PDB ID 4UPB) (5). FrdABCD are colored by subunits. ViaA is colored by its domains. RavA hexamer is shown in one color. The RavA-LdcI complex consists of two LdcI decamers and five RavA hexamers. Each LdcI decamer consists of two pentamers that are colored in red and grey each, while the five RavA hexamers are colored in different shades of blue. It should be noted that, it is not yet established whether ViaA binds to RavA when RavA forms a complex with LdcI.

**REFERNCES**

1. Ishida, T., and Kinoshita, K. (2007) PrDOS: prediction of disordered protein regions from amino acid sequence. *Nucleic Acids Res.* **35**, W460-464

2. Shen, Y., Delaglio, F., Cornilescu, G., and Bax, A. (2009) TALOS+: a hybrid method for predicting protein backbone torsion angles from NMR chemical shifts. *J. Biomol. NMR* **44**, 213-223

3. Iverson, T. M., Luna-Chavez, C., Croal, L. R., Cecchini, G., and Rees, D. C. (2002) Crystallographic studies of the Escherichia coli quinol-fumarate reductase with inhibitors bound to the quinol-binding site. *J. Biol. Chem.* **277**, 16124-16130

4. Jessop, M., Arragain, B., Miras, R., Fraudeau, A., Huard, K., Bacia-Verloop, M., Catty, P., Felix, J., Malet, H., and Gutsche, I. (2020) Structural insights into ATP hydrolysis by the MoxR ATPase RavA and the LdcI-RavA cage-like complex. *Commun Biol* **3**, 46

5. Malet, H., Liu, K., El Bakkouri, M., Chan, S. W., Effantin, G., Bacia, M., Houry, W. A., and Gutsche, I. (2014) Assembly principles of a unique cage formed by hexameric and decameric E. coli proteins. *Elife* **3**, e03653
